# Multistate Outbreak of Listeriosis Linked to Soft-Ripened Cheese — United States, 2013

**Published:** 2014-04-04

**Authors:** Mary J. Choi, Kelly A. Jackson, Carlota Medus, Jennifer Beal, Carrie E. Rigdon, Tami C. Cloyd, Matthew J. Forstner, Jill Ball, Stacy Bosch, Lyndsay Bottichio, Venessa Cantu, David C. Melka, Wilete Ishow, Sarah Slette, Kari Irvin, Matthew Wise, Cheryl Tarr, Barbara Mahon, Kirk E. Smith, Benjamin J. Silk

**Affiliations:** 1 Minnesota Department of Health; 2 EIS officer; 3 Div of Foodborne, Waterborne, and Environmental Diseases, National Center for Emerging and Zoonotic Infectious Diseases, CDC; 4 Coordinated Outbreak Response and Evaluation Network, Food and Drug Administration; 5 Minnesota Department of Agriculture; 6 Wisconsin Department of Agriculture, Trade, and Consumer Protection; 7 Ohio Department of Health; 8 Texas Department of State Health Services; 9 Center for Food Safety and Applied Nutrition, Food and Drug Administration; 10 Chicago Department of Public Health; 11 Indiana State Department of Health

On June 27, 2013, the Minnesota Department of Health notified CDC of two patients with invasive *Listeria monocytogenes* infections (listeriosis) whose clinical isolates had indistinguishable pulsed-field gel electrophoresis (PFGE) patterns. A query of PulseNet, the national molecular subtyping network for foodborne disease surveillance, identified clinical and environmental isolates from other states. On June 28, CDC learned from the Food and Drug Administration’s Coordinated Outbreak Response and Evaluation Network that environmental isolates indistinguishable from those of the two patients had been collected from Crave Brothers Farmstead Cheese during 2010–2011. An outbreak-related case was defined as isolation of *L. monocytogenes* with the outbreak PFGE pattern from an anatomic site that is normally sterile (e.g., blood or cerebrospinal fluid), or from a product of conception, with an isolate upload date during May 20–June 28, 2013. As of June 28, five cases were identified in four states (Minnesota, two cases; Illinois, Indiana, and Ohio, one each). Median age of the five patients was 58 years (range: 31–67 years). Four patients were female, including one who was pregnant at the time of infection. All five were hospitalized. One death and one miscarriage were reported.

Case–case analysis of *Listeria* Initiative* data (1) was conducted, comparing food exposure frequencies among the five outbreak-related cases identified by June 28 with food exposure frequencies in 1,735 sporadic listeriosis cases reported to CDC during 2004–2013. The analysis indicated that any soft cheese consumption during the month before illness onset was associated with outbreak-related listeriosis: five of five (100%) in the outbreak-related cases versus 569 of 1,735 (33%) in the sporadic cases (odds ratio = 10.8; 95% confidence interval = 1.8−∞).

The five patients were reinterviewed to assess their cheese exposures. All five patients had definitely or probably eaten one of three varieties of Crave Brothers soft-ripened cheese (Les Frères, Petit Frère, or Petit Frère with truffles). Three patients had purchased the cheese at three different restaurants, and two had purchased the cheese at two different grocery stores. The cheeses were shipped as intact wheels to the three restaurants and two grocery stores, where they had been cut and served or repackaged and sold to customers.

Testing at the Minnesota Department of Agriculture identified the outbreak pattern of *L. monocytogenes* in two cheese wedges (Les Frères and Petit Frère with truffles) collected from two different grocery stores in Minnesota. Inspection of the cheese-making facility revealed that substantial sanitation deficiencies during the cheese-making process itself, after the milk was pasteurized, likely led to contamination. On July 1, Crave Brothers halted production of Les Frères, Petit Frère, and Petit Frère with truffles. On July 3, Crave Brothers issued a voluntary recall of these products with a production date of July 1, 2013, or earlier. On July 11, the company voluntarily halted production of all cheese products manufactured at the facility. After product recall, one additional case was identified in Texas through whole genome sequencing, bringing the total case count for the outbreak to six.

This outbreak was linked to soft cheeses that were likely contaminated during the cheese-making process (2,3). Pasteurization eliminates *Listeria* in milk. However, contamination can occur after pasteurization. Cheese-making facilities should use strict sanitation and microbiologic monitoring, regardless of whether they use pasteurized milk.†

Persons at greater risk for listeriosis, including older adults, pregnant women, and those with immunocompromising conditions, should be aware that certain soft cheeses made with unpasteurized milk, or made under unsanitary conditions, regardless of whether the milk was pasteurized, have been shown to cause severe illness. These soft cheeses include fresh (unripened) cheeses, such as queso fresco (4), and soft-ripened cheeses, such as the cheeses implicated in this outbreak.

## Footnotes

* The *Listeria* Initiative is an enhanced surveillance system that has routinely collected data regarding food consumption from all patients with listeriosis since 2004. Additional information is available at http://www.cdc.gov/listeria/pdf/listeriainitiativeoverview_508.pdf.

† Joint Food and Drug Administration/Health Canada quantitative assessment of the risk of listeriosis from soft-ripened cheese consumption in the United States and Canada: draft report. Available at http://www.fda.gov/downloads/food/foodscienceresearch/ucm338617.pdf.
